# Deep Learning for Acute Myeloid Leukemia Diagnosis

**DOI:** 10.25122/jml-2019-0090

**Published:** 2020

**Authors:** Elham Nazari, Amir Hossein Farzin, Mehran Aghemiri, Amir Avan, Mahmood Tara, Hamed Tabesh

**Affiliations:** 1. Department of Medical Informatics, Faculty of Medicine, Mashhad University of Medical Sciences, Mashhad, Iran; 2. Department of Computer Engineering, Khayyam University, Mashhad, Iran; 3. Department of Medical Informatics, Faculty of Medical Sciences, Tarbiat Modares University, Tehran, Iran; 4. Molecular Medicine Group, Department of Modern Sciences and Technologies, School of Medicine, Mashhad University of Medical Sciences, Mashhad, Iran

**Keywords:** AML, machine learning, deep learning, neural network, microarray

## Abstract

By changing the lifestyle and increasing the cancer incidence, accurate diagnosis becomes a significant medical action. Today, DNA microarray is widely used in cancer diagnosis and screening since it is able to measure gene expression levels. Analyzing them by using common statistical methods is not suitable because of the high gene expression data dimensions. So, this study aims to use new techniques to diagnose acute myeloid leukemia.

In this study, the leukemia microarray gene data, contenting 22283 genes, was extracted from the Gene Expression Omnibus repository. Initial preprocessing was applied by using a normalization test and principal component analysis in Python. Then DNNs neural network designed and implemented to the data and finally results cross-validated by classifiers.

The normalization test was significant (P>0.05) and the results show the PCA gene segregation potential and independence of cancer and healthy cells. The results accuracy for single-layer neural network and DNNs deep learning network with three hidden layers are 63.33 and 96.67, respectively.

Using new methods such as deep learning can improve diagnosis accuracy and performance compared to the old methods. It is recommended to use these methods in cancer diagnosis and effective gene selection in various types of cancer.

## Introduction

Big medical data generated as a result of recent advances in biology. Using appropriate analyzing methods led biologists to percept the complex dynamic system of life. It is one of the most critical challenges for biologists. Microarray is well known despite it is a new technology in molecular biology. It is used to monitoring genome-wide expression levels by biologists [1]. This technology includes examining a thousand genes and protein activity on a small scale to compare the similarity and track changes such as track decreasing or increasing gene activities and track protein samples changes in comparison to the control sample [2]. Microarray uses include genotyping, epigenetics, translation profiling, gene expression profiling [3]. Microarray can be replaced by aggressive cancer detection methods such as bone marrow biopsy, which is used in the accurate diagnosis of acute myeloid leukemia. Bone marrow biopsy is invasive, painful, and can cause serious complications such as infections and bleeding [4]. Therefore, microarrays improve clinical diagnosis providing high accuracy diagnostic procedures. It can be used as a gold standard to diagnose [5] and help to treatment progress development and understanding cell biology, especially in oncology studies. Thus, the gene expression patterns are compared in two healthy and cancerous tissues. Cancer is closely linked to genetic changes [6]; hence, the accurate cancer diagnosis speeds up by using a pattern to classify normal and cancer cells [7]. So, a timely and accurate diagnosis is essential.

There are many pieces of evidence that accurate cancer diagnosis is one of the most effective ways of reducing the mortality rate [7,8]. The higher number of genes (p) compare to the number of tissues (n) is the feature of the microarray [9]. It is challenging to analyze microarray data using statistical methods to classify high-dimensional data (p>n) due to overestimation and multiple linearity problems make statistical classification of microarrays difficult [10, 11]. Its analyzing methods are evolving rapidly, and there is no specific way considered the best way to analyze microarrays [2]. Recently, expert systems to diagnose cancerous gene data are increasing, and machine learning techniques are currently used more. Machine learning can help to automation and intelligence process, improve development, accuracy and reducing costs [12]. Machine learning, ensemble methods, and deep learning are showing high performance in classifying biological data [13-16].

In this study, neural networks and deep learning were used to separate healthy and cancerous cells in leukemia related genes. Acute myeloid leukemia (AML) is the type of cancer that starts in the bone marrow, but in most of the cases, it moves to the blood very fast. This type of cancer worsens fast if left untreated [17].

## Material and Methods

In this study, we classified healthy and cancerous cells by neural networks and deep learning. 

### Artificial neural network

It is a computational and algorithmic model inspired by the structure and functional aspects of biological neural networks and the concept of neurons. It is considered one of the nonlinear statistical data modeling tools and is used for pattern recognition and modeling complex relations between inputs and outputs. It consists of some simple units that work in parallel. Weighting between units is the primary way to store information long-term and learn new information by updating weights. 

**Figure 1:**
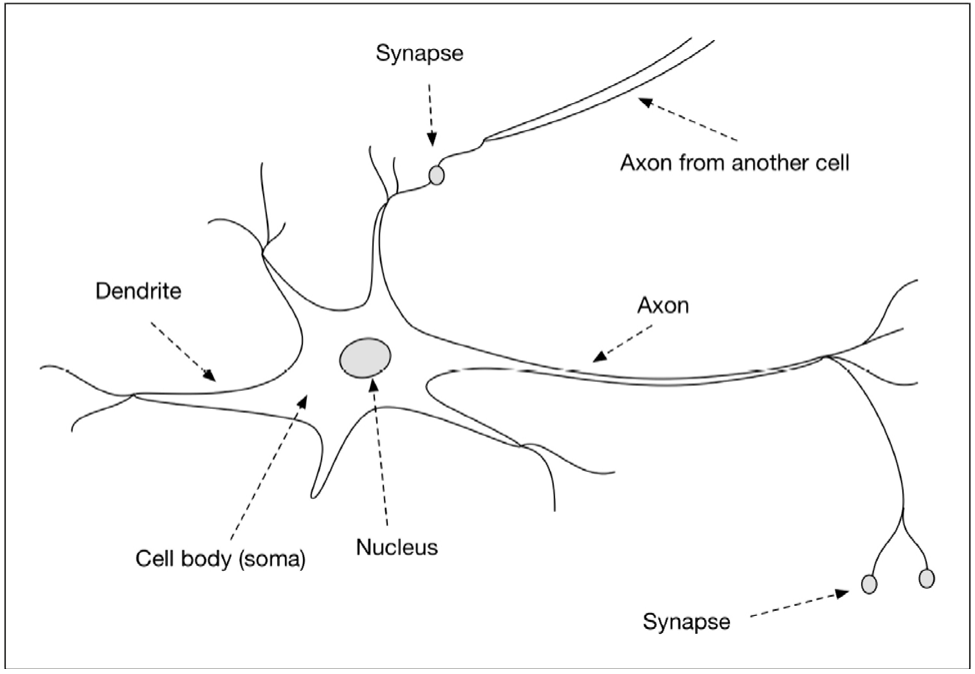
Structure of a typical neuron.

A neuron of the human nervous system consists of dendrites, a single axon, soma, and nucleus, as shown in Figure 1.

**Figure 2:**
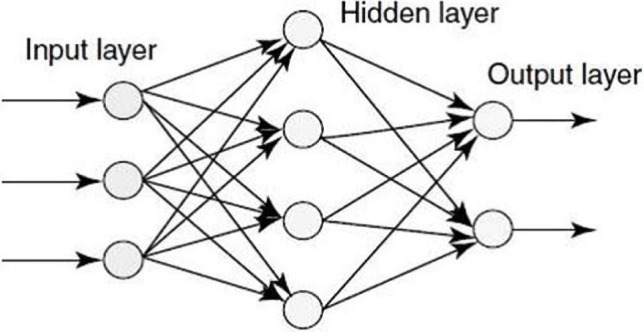
Structure of an artificial neural network.

Dendrite receives electrochemical impulses from the other neurons. The soma processes these signals. The output is transmitted to terminal dendrites by axons, where these new impulses are sent to the next neuron. An artificial neural network works the same way on three layers: the input layer gets data (dendrite), the hidden layer processes data (soma and axon), and finally, processed data is sent to the output layer (synapse)(Figure 2) [18-21].

The neural network’s behavior is shaped by the architecture of that network. Neural network architecture can be defined as follows:

• The number of neurons;
• Number of layers;
• Types of communication between layers.

Perceptron is one of the simplest neural networks. It is a learning algorithm for a binary classifier, called a threshold function:


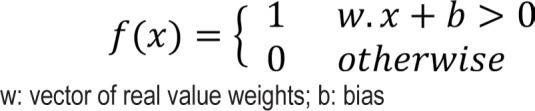


**Figure 3:**
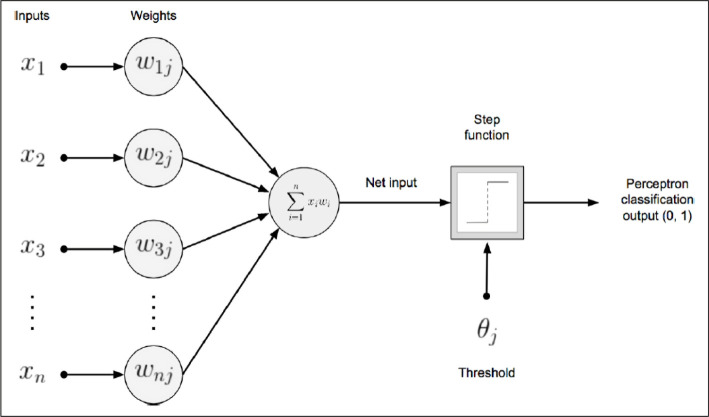
A single-layer perceptron.

The main configuration of perceptron networks is shown in Figure 3.

Activation functions are used to propagate the node outputs from one layer to the next (up to the output layer). The activation function is a function that activates the neuron. There are several types of activation functions such as Identity, Binary Step, Sigmoid, Tanh, ReLU, Leaky ReLU, and Softmax. 

Sigmoid is a widely used activation function converting illimitable independent variables to simple probabilities between 0 and 1. Sigmoid can infinitely reduce data or outlying values without deleting them. Unlike the sigmoid activation function, Tanh is bound to the (-1,1) range. It is worth mentioning that tanh deals easier with negative numbers. Besides, tanh is a well-liked and widely used activation function. The Softmax function is a multiclass logistic regression and a generalization of the sigmoid. Therefore, it can be applied to continuous data (rather than binary classification). Rectified linear units (ReLU) are based on the latest scientific advances, and it has been proven to be working in many conditions. ReLU is bound to the [0, inf) range. It makes the network lighter and efficient due to the characteristics of ReLU. Also, these activation functions show better performance than Sigmoid in training data. Recent studies show that deep learning networks using ReLU are able to train well without preprocessing techniques.

Loss functions determine how much a trained neural network is close to reality. It is a measured inconsistency between the real and predicted value considered as an error; the mean error value takes and represents the difference between the real-world and the neural network. There are many common loss functions, such as MAE, MAPE, MSLE, and MSE [19].

### Learning rate

It determines the neural network values and how much they change by new training data. The learning rate is set before the learning process begins. A low learning rate means more time to train, but a high learning rate makes the network more sensitive to new information [20, 21].

### Deep learning

Recently, a new machine learning technique known as deep learning, is used frequently. New studies show that this algorithm has better results compared to machine learning, for example, identifying and discovering drugs, image processing, and speech [22-27].

Deep-learning is defined as a neural network with a large number of parameters and layers. In fact, it is a class of machine learning algorithms that uses a hierarchical nonlinear structure in multiple layers to extract features and transformations [19].

Unlike other machine learning methods requiring an expert to extract features, deep learning can act as an automatic feature extractor that transforms low-level features into higher-level abstractions [28]. In addition, deep learning can incorporate momentary, indirect and minor changes and leads to higher accuracy than other machine learning methods [29].

Types of deep learning techniques can include deep neural networks (DNNs), autoencoders networks (AEs), generative adversarial networks (GANs), repeating neural networks (RNNs), convolutional neural networks (CNNs) and more [19].

### Quality control

This is one of the steps of the microarray data analyzing, after which it is possible to test and interpret the method.

Any negligence to impose quality control may cause detour and alter the results significantly for many reasons, such as the following:

1. The biologist grows the cell culture without knowing that bacteria may live in the cell.
2. There may be fungal or viral contamination.
3. The RNA treatment may not do well after RNA extraction.
4. Because RNA is a highly unstable molecule and it begins to crumble, the quality decreases at room temperature.
5. The sample size is not enough, or there is an error in complementary DNA (cDNA) generation in rank steps.
6. The results are not reliable if something goes wrong during the scanning or hybridization steps.

Biases that occur in the study results related to genetic data lead to false-positive and false-negative results.

The genes that can separate the cancerous and healthy cells indicate that the experiment is well done. So, dimension reduction techniques are used to detect important genes in separating these samples. Otherwise, co-expression or co-relation between genes or between samples can be measured. Actually, the purpose of dimension reduction is to capture the variations in microarray data [30, 31].

### PCA

Principal component analysis (PCA) is an analysis of simplifying high-dimensional complexity, including patterns and trends. High-dimensional data is common in biology, and multiple features occur when the expression of the different genes for each sample is measured [32, 33].

### Dataset

In this study, we used the Gene Expression Omnibus (GEO) database and AML-related data, and healthy and cancerous cases were extracted. GEO is a public and international database that publishes free genomic data obtained from microarray studies, Next Generation Sequencing (NGS), sequence-based functional genomics studies, and handles high-throughput data submissions.

Data from 36 cases containing 22283 gene expression of AML microarray experiments were retrieved from the GSE9476 dataset, including 10 healthy and 26 AML cases. The dataset was registered in 2007 and updated in 2017.

### Data preparation

Microarray data normalization is one of the essential microarray data analyzing steps, balancing the hybridization intensity of each point in the data matrix. Comparability of samples was examined by Python 3.7 and data normality was confirmed [34].

In this study, the PCA method was used for dependency testing and data quality.

## Results

### Modeling

Normalized data modeled by TensorFlow, Pandas, Numpy, Sklearn packages, and activity functions include Softmax, Adamax, and RELU. These packages explained the following.

In this study, we used three hidden layers of DNNs to analyze the data. Due to using dropout, the overfitting rate was reduced, the Adamax function was used as an optimizer, and sparse categorical was used to calculate the loss. Accuracy was considered as the network improvement criterion at each epoch. Also, 70% of data was used as training data, and the number of epochs was considered 1000.

TensorFlow is an open-source library for large-scale numerical computing and machine learning techniques, making it easier and faster to apply machine learning and deep learning techniques.

Pandas provides fast, flexible and illustrative data structures in Python. It also enables data entry, manipulation, and data analysis.

Numpy is an open-source package that supports scientific calculations, matrices, and multidimensional arrays. It also supports functions such as Sine, Cose, Log, and others.

Sklearn is an open-source package in Python that offers powerful data analysis and data mining tools.

### Analysis of results related to AML data

**Figure 4:**
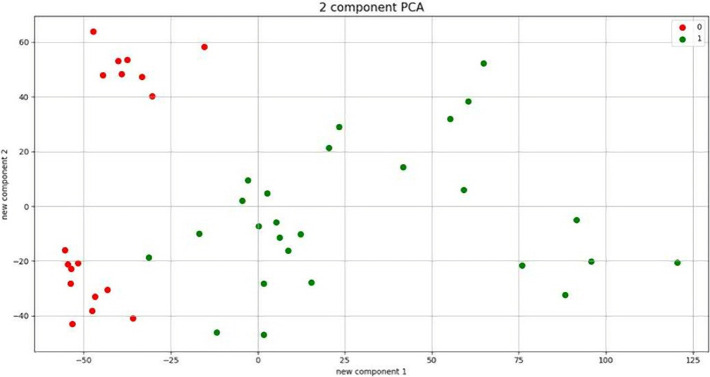
PCA for microarray data.

The PCA result is shown in Figure 4.

As can be seen, the cancerous and healthy data were separated, indicating that the experiment was performed correctly and had the prerequisite to implement the method, and the results are reliable. The result of a simple neural network and the deep learning-based network implementation is shown below: 

A simple neural network with a middle layer has an accuracy of 0.6333, and a deep learning network with three hidden layers has an accuracy of 0.9667

According to the results, neural networks based on deep learning techniques provided higher accuracy than a simple neural network.

## Discussion

Microarray technology has enabled thousands of genes to be analyzed simultaneously and, very important, in the early detection of diseases, including cancer. It is difficult to analyze these types of data by usual techniques because of their high dimensionality. Therefore, in this study, deep learning-based techniques and its comparing simple neural network were used to detect AML. The reported result shows higher accuracy in the classification of cancerous and healthy tissues by a deep learning-based network. 

Deep learning methods are a class of machine learning techniques able to identify patterns with high complexity in large datasets, and it can analyze data with large numbers of features and samples with high accuracy. The AML dataset had 22283 features, so it was used for modeling by deep learning [35, 36]. Today, this is a state of the art of machine learning techniques. The high performance of these types of machine learning methods in various industries, including healthcare, medicine and bioinformatics, has been confirmed [35]. This technique presents valuable results in various medical fields such as thyroid diagnosis using images [37], heart disease detection [38], breast cancer diagnosis [39, 40], molecular properties identification of drugs damaging the liver [41], orally disintegrating tablets (ODT) formulation prediction using an artificial neural network [42], and predicting water solubility of medication using other machine learning methods [43]. In addition, it has been exploited in modeling the sequence specificity of DNA–protein binding [44], genes prediction [45], motif identification, binding classification [46], protein binding [47], predicting genomic sequence, and the effects of non-coding variants [48]. Finding genes is the most crucial research problem in bioinformatics. Researchers have proposed different models for finding genes in the DNA sequence, but sometimes it does not work because of DNA sequence length variety and low accuracy [49].

Further studies with this aim are recommended in the future. Also, omic data are beneficial for discovering drugs and biomarkers. These data are highly variable, high-dimensional, and use multiple sources. 

## Conclusion

Deep neural networks are efficient algorithms using compositional layers of neurons to overcome omic data challenges [50]. In future studies, deep learning-based techniques for medical image processing, genetic data, audio data, handwriting recognition for diagnosis and therapies are recommended due to high performance and the ability to manage complexity and data variations.

## Acknowledgments

The present study results from a research project approved by the vice chancellery for research of the Mashhad University of Medical Sciences (Grant number 961731).

## Conflict of Interest

The authors declare that there is no conflict of interest.

## References

[1] Babu MM. (2004). Introduction to microarray data analysis. Computational genomics: Theory and application.

[2] Akay M-F (2009). Support vector machines combined with feature selection for breast cancer diagnosis. Expert Syst Appl.

[3] Shaik NA., Hakeem KR, Banaganapalli B, Elango R (2019). Essentials of Bioinformatics, Volume I: Understanding Bioinformatics: Genes to Proteins.

[4] PERCIVAL Mary-Elizabeth (2017). Bone marrow evaluation for diagnosis and monitoring of acute myeloid leukemia. Blood reviews.

[5] Yoo S., Choi J, Lee S, Yoo N (2008). Applications of DNA microarray in disease diagnostics. J Microbiol Biotechnol.

[6] Sack G-H (1999). Medical Genetics.

[7] Tarver Talicia (2012). Cancer facts & figures 2012. American cancer society (ACS).

[8] Curry Susan J. (2003). Potential of screening to reduce the burden of cancer. Fulfilling the Potential of Cancer Prevention and Early Detection.

[9] Kalina J. (2014). Classiﬁcation methods for high-dimensional genetic data. Biocybern Biomed Eng.

[10] Piao Y., Piao M, Park K, Ryu K-H (2012). An ensemble correlation-based gene selection algorithm for cancer classiﬁcation with gene expression data. Bioinformatics.

[11] Chen K., Wang K, Wang K, Angelia M (2014). Applying particle swarm optimization-based decision tree classiﬁer for cancer classiﬁcation on gene expression data. Appl Soft Comput.

[12] Rowe RC., Roberts RJ (1998). Artificial intelligence in pharmaceutical product formulation: knowledge-based and experts ystems. Pharm SciTechnol Today.

[13] Cancer is the second leading cause of death globally (2018). http://www.who.int/news-room/fact-sheets/detail/cancer.

[14] Liu KH., Tong M, Xie ST, Yee Ng VT (2015). Genetic programming based ensemble system for microarray data classification. Computational and mathematical methods in medicine.

[15] Tan AC., Gilbert D Ensemble machine learning on gene expression data for cancer classification.

[16] Yue T., Wang H (2018). Deep learning for genomics: A concise overview. arXiv preprint arXiv:1802.00810.

[17] Rubnitz JE., Gibson B, Smith FO (2008). Acute myeloid leukemia. Pediatric clinics of North America.

[18] Dey A. (2016). Machine learning algorithms: a review. International Journal of Computer Science and Information Technologies.

[19] Patterson J., Gibson A (2017). Deep learning: A practitioner’s approach.

[20] Sharma V., Rai S., Dev A. (2012). “A Comprehensive Study of Artificial Neural Networks”. International Journal of Advanced Reasearch in Computer Science and Software Engineering.

[21] Hiregoudar S. B., Manjunath K., Patil K. S. (2014). “A Survey: Research Summary on Neural Networks”. International Journal od Research in Engineering and Technology.

[22] Lusci A, Pollastri G, Baldi P (2013). Deeparchitecturesanddeeplearningin chemo informatics :thepredictionofaqueoussolubilityfordrug-like molecules. J ChemInfModel.

[23] Ma J, Sheridan RP, Liaw A, Dahl GE, Svetnik V (2015). Deepneuralnetsasmethodforquantitativestructure–activity relationships. J ChemInf Model.

[24] Altae-Tran H, Ramsundar B, Pappu AS, Pande V (2017). Lowdatadrug discovery with one-shot learning. ACS CentSci.

[25] The Next Era: Deep Learning in Pharmaceutical Research Sean Ekins

[26] Yang Yilong (2018). Deep learning for in vitro prediction of pharmaceutical formulations.

[27] Han Run, Yang Yilong, Li Xiaoshan, Ouyang Defang, Predicting oral disintegrating tablet formulations by neural network techniques

[28] Schmidhuber J (2015). Deep learning in neural networks: an overview. Neural Netw.

[29] Rost B, Sander C (1994). Combining evolutionary information and neural networks to predict protein secondary structure. Proteins.

[30] Raman T., O’Connor TP, Hackett NR, Wang W, Harvey BG, Attiyeh MA, Dang DT, Teater M, Crystal RG (2009). Quality control in microarray assessment of gene expression in human airway epithelium. BMC genomics.

[31] Anderson CA., Pettersson FH, Clarke GM, Cardon LR, Morris AP, Zondervan KT (2010). Data quality control in genetic case-control association studies. Nature protocols.

[32] Jolliffe I. (2011). Principal component analysis.

[33] Raychaudhuri S., Stuart JM, Altman RB (1999). Principal components analysis to summarize microarray experiments: application to sporulation time series. InBiocomputing 2000.

[34] Quackenbush J. (2002). Microarray data normalization and transformation. Nat Genet.

[35] Min S., Lee B, Yoon S (2017). Deep learning in bioinformatics. Briefings in bioinformatics.

[36] Zou J., Huss M, Abid A, Mohammadi P, Torkamani A, Telenti A (2018). A primer on deep learning in genomics. Nature genetics.

[37] Ma L., Ma C, Liu Y, Wang X (2019). Thyroid diagnosis from SPECT images using convolutional neural network with optimization. Computational intelligence and neuroscience.

[38] Tomov NS., Tomov S (2018). On Deep Neural Networks for Detecting Heart Disease. arXiv preprint arXiv:1808.07168.

[39] Mohamed AA., Berg WA, Peng H, Luo Y, Jankowitz RC, Wu S (2018). A deep learning method for classifying mammographic breast density categories. Medical physics.

[40] Abdel-Zaher AM., Eldeib AM (2016). Breast cancer classification using deep belief networks. Expert Systems with Applications.

[41] Xu Y., Dai Z, Chen F, Gao S, Pei J, Lai L (2015). Deep learning for drug-induced liver injury. Journal of chemical information and modeling.

[42] Han R., Yang Y, Li X, Ouyang D (2018). Predicting oral disintegrating tablet formulations by neural network techniques. Asian Journal of Pharmaceutical Sciences.

[43] Lusci A., Pollastri G, Baldi P (2013). Deep architectures and deep learning in chemoinformatics: the prediction of aqueous solubility for drug-like molecules. Journal of chemical information and modeling.

[44] Zeng H., Edwards MD, Liu G, Gifford DK (2016). Convolutional neural network architectures for predicting DNA–protein binding. Bioinformatics.

[45] Sree PK., Rao PS, Devi NU CDLGP: A novel unsupervised classifier using deep learning for gene prediction.

[46] Lanchantin J., Singh R, Lin Z, Qi Y (2016). Deep motif: Visualizing genomic sequence classifications. arXiv preprint arXiv:1605.01133.

[47] Zeng H., Edwards MD, Liu G, Gifford DK (2016). Convolutional neural network architectures for predicting DNA–protein binding. Bioinformatics.

[48] Yue T., Wang H (2018). Deep learning for genomics: A concise overview.

[49] Sree PK., Rao PS, Devi NU CDLGP: A novel unsupervised classifier using deep learning for gene prediction.

[50] Mamoshina P., Vieira A, Putin E, Zhavoronkov A (2016). Applications of deep learning in biomedicine. Molecular pharmaceutics.

